# The Measurement of Food Insecurity in High-Income Countries: A Scoping Review

**DOI:** 10.3390/ijerph18189829

**Published:** 2021-09-17

**Authors:** Elena Carrillo-Álvarez, Blanca Salinas-Roca, Lluís Costa-Tutusaus, Raimon Milà-Villarroel, Nithya Shankar Krishnan

**Affiliations:** 1 Global Research on Wellbeing (GRoW) Research Group, Blanquerna School of Health Science, Ramon Llull University, Padilla, 326-332, 08025 Barcelona, Spain; blancasr@blanquerna.url.edu (B.S.-R.); lluisct@blanquerna.url.edu (L.C.-T.); raimonmv@blanquerna.url.edu (R.M.-V.); nithya.krishnan@outlook.com (N.S.K.); 2 Department of Nursing and Physiotherapy, University of Lleida, Montserrat Roig 2, 25198 Lleida, Spain

**Keywords:** food insecurity, measurement, instruments, high-income countries, scoping review

## Abstract

The measurement of food insecurity is essential to monitor the prevalence, risk factors, consequences and effects of food insecurity and the interventions and policies implemented to tackle it. Yet, how best to apply it remains an unsettled issue due to the multifaceted and context-dependent nature of food insecurity. We report a scoping review of measures of food insecurity at the individual and household level in high-income countries with the final purpose of facilitating a catalogue of instruments to be used by both researchers and practitioners. The scoping review was conducted following the methodological framework of Arksey and O’Malley and the Joanna Briggs Institute guidelines. We included all types of documents published between 2000–2020 using instruments that estimate food insecurity at both individual and household level in high-income countries, and with respondents including adolescents, adults, and elderly. We identified a total of 23 measurement strategies being used in 33 peer-reviewed publications and 114 documents from the grey literature. Our results show that most measures focus on the access dimension of food insecurity and that further research is required to develop measures that incorporate aspects of quality of dietary intake and relevant individual, household and social conditions related to food insecurity.

## 1. Introduction

The 2020 edition of the Food and Agriculture Organization’s (FAO) yearly report “The State of Food Security and Nutrition in the World (SOFI)” showed that food insecurity was rising. Worldwide, it was estimated that in 2019, 750 million people (9.7%) were exposed to severe levels of food insecurity and more than 3 billion people could not afford a healthy diet as a consequence of conflict and civil strife, climate variability, economic crisis, and persistent poverty and inequality [1]. The COVID-19 situation has added a further threat to food security across the globe, as a result of which 148 million additional people became severely food insecure [2]. According to the same report, less than 2.5% of the population in Europe and North America would be in this situation, but moderate and severe food insecurity levels have risen to almost 9%, getting closer to 2014 levels. More localized analysis shows that prevalences of food insecurity in specific populations of these territories could be above 10–15% [3,4,5,6].

Food insecurity threatens both individuals and societies, and constitutes a serious threat to public health and wider society [7]. In children, it has been associated with impaired physical and psychological development, the effects of which can persist into adolescence and adulthood. In adults, characteristic conditions of the double burden of diseases have been observed, such as the combination of nutritional deficiencies (i.e., anemia) and excess-diseases (i.e., diabetes, obesity). In addition, food insecurity can result in psychological burden and social stigma [8,9], and can also have socioeconomic spillovers in terms of productivity and health-care expenditure. People’s experiences and how they cope with food insecurity have been extensively studied and documented by sociologists and anthropologists, and have considerably influenced our comprehension of what food insecurity entails [10,11]

The concept, interest in and understanding of food insecurity has evolved since it was first coined by the World Bank as a notion designated to broaden the understanding of hunger. The first definition of food security, “access by all people at all times to enough food for an active, healthy life” [12], was expanded ten years later in the World Food Summit to include recognition of the multidimensionality of the concept. Since then, food security is mostly referred to as the situation in which “all people at all times have physical, economic and social access to sufficient, safe and nutritious food to maintain a healthy and active life” [13]. Additionally, the Committee on World Food Security added in 2012 that ‘it is supported by an environment of adequate sanitation, health services and care, allowing for a healthy and active life’ [14], something that has been proven crucial [15].

The interest in hunger and food insecurity was initially in low-income (then referred to as developing) countries. However, it gathered momentum in high-income (developed) countries thanks to Kathy Radimer’s seminal work at Cornell University [16], where she explored the phenomena and experiences of food insecurity in the US. Drawing on her findings and those of other scholars, the USDA adopted “the limited or uncertain availability of nutritionally adequate and safe foods, or limited or uncertain ability to acquire acceptable foods in socially acceptable ways” [17] as the definition of food insecurity, incorporating elements of contextualization to the definition of food (in)security, e.g., what is acceptable in one sociocultural environment may not be in another.

These remarks on the definitions are significant since the measurement of food insecurity draws on them. As mentioned, the definition provided by the FAO is the most commonly used and comprises of four dimensions that are necessary to achieve food security: availability, access, utilization and stability [18]. Availability refers to the supplies that are sourced for individuals to cover their dietary needs. Therefore, it is determined by food production, stock levels and net trade, and generally considered at the country-level and assessed through balance sheets. Access concerns how individuals reach the food available in their environment and is in turn conditioned by physical (i.e., transportation) and economic (i.e., prices, incomes) constraints. It is normally assessed at the household or individual level, and three domains of inadequate household-level food access are distinguished: quantity, quality and the psychological effects of inadequate quantity or quality food supply [19]. Utilization involves ensuring proper nutrition for the organism, and comprises elements such as intra-household distribution, purchase and conservation practices, food preparation, etc. As such, its measurement is generally done at the individual level. In order to achieve food security, all the former dimensions need to occur simultaneously and be stable over time. In high-income countries, the biggest constraint is found in the access dimension, and it is typically addressed through social wages, food banks, and soup kitchens [20].

The measurement of food insecurity is essential to monitor the prevalence, risk factors, consequences and effects of food insecurity and the interventions and policies implemented to tackle it. Yet, how best to apply it remains an unsettled issue mainly due to the multifaceted and context-dependent nature of food insecurity [16,19,21,22]. In recent years, several reviews of food insecurity measurement in high-income countries have been published in the literature [16,21,22,23,24,25,26,27,28]. With some differences in the objective of their study, databases and date of the search, as well as data retrieved, their results show how currently food insecurity is mostly assessed through multiple item questionnaires. However, these can be long, burdensome to respond and costly to implement. Moreover, most measures evaluate the access dimension of food insecurity and do not adequately capture its psychological and quality domains, nor the experience of or consequences for individuals. An additional question refers to the appropriateness of using tools developed for low- and middle-income countries in high-income ones, and vice versa. In view of the alarming rates of food insecurity in high-income countries, it is necessary to identify the most adequate tools to measure it, considering the nature, characteristics, and requirements of food insecurity in these contexts.

We aim at complementing and expanding these former works through a scoping review of measures of food insecurity at the individual and household level in high-income countries, which will also include the grey literature, with the final purpose of facilitating a catalogue of instruments to be used by both researchers and practitioners.

Hence, this review delves into the various types of tools that have been employed to measure food insecurity in high-income countries at the individual and household level, focusing on the following research questions: (i) What are the existent instruments available to measure food insecurity in individuals and households in high-income countries? (ii) What dimensions of food insecurity are the ones more often included and the ones that are missing? (iii) What are the typical characteristics of the instrument?, i.e., scale’s origin (country, institution, and language), number of items, types of response options, psychometric features of the instrument, focus on quantity or/and quality of food, number of articles using the particular instrument, etc.? (iv) What are the differences in the measures used for different target groups based on age, i.e., adolescents, adults and elderly?

## 2. Materials and Methods

This review was conducted following the methodological framework of Arksey and O’Malley [29] and the Joanna Briggs Institute guidelines [30]. These frameworks involve key phases which include identifying the research question; identifying relevant studies; study selection; charting data; and finally collating, summarizing, and reporting results. In this case, we have not pursued the sixth phase of stakeholder consultation. Consequently, the Guidelines published by the Joanna Briggs Institute as well as the Preferred Reporting Items for scoping reviews (PRISMA-ScR) and the Preferred Reporting Items for Systematic Reviews and Meta-analysis Protocols Statement (PRISMA-P) have been also followed [31,32].

### 2.1. Eligibility Criteria, Information Sources and Search Strategy

The eligibility criteria include the following: (i) original studies, review articles, food aid and food insecurity reports published in peer reviewed journals as well as the grey literature—which include reports and statistics from government sources and NGOs, dissertations or theses, conferences and presentations, and newsletters and press articles- reporting to have used (ii) instruments that estimate food insecurity in adults at both individual and household level (iii) in high-income countries according to the World Bank [33] and with (iv) respondents including adolescents, adults and elderly. Papers reporting food insecurity prevalence or its health effects in children were excluded. No language restrictions were established, and the timeframe for inclusion was 2000–2020.

The search strategy for information sources was devised in consultation with an academic librarian along with two members of the research team with expertise in review methodology. The algorithms for search strategy focused on title, keywords and abstract. Key words included: “food insecurity”, “food security”, “dietary diversity”, “food insufficiency”, “food poverty”, “measurement” OR “measure”, “instrument”, “scale”, “questionnaire”, “high-income countries”, “developed countries”, “individual”, “household” (….). Specific searches for each database can be found in Supplementary Materials File S1.

For the peer-reviewed literature, the following medical and social science bibliographic databases were searched: Cochrane Central Register of Controlled Trials (CENTRAL), Medline (PubMed), Embase, CINAHL, FSTA, PsycINFO, Scopus, Sociological Abstracts and Web of Science Core Collection. The reference list of the eligible papers was scanned for additional registries.

The search for the grey literature was conducted by two research team members. Since there is not yet a gold standard for the grey literature, the search was carried out mainly via search engines such as Google, Google Scholar and OpenGray. Specific steps and recommended resources for searching the grey literature were followed [34]. To ensure that a large amount of the of grey literature was included, the search was also extended to specific websites which provided information on food insecurity such as FAO, USDA, UNICEF, WHO, etc. In addition, top university websites of the world offering a comprehensive list of grey literature databases such as DOAR, ELDIS, Factiva, periodicals index online were also searched.

### 2.2. Study Selection

**Figure 1:**
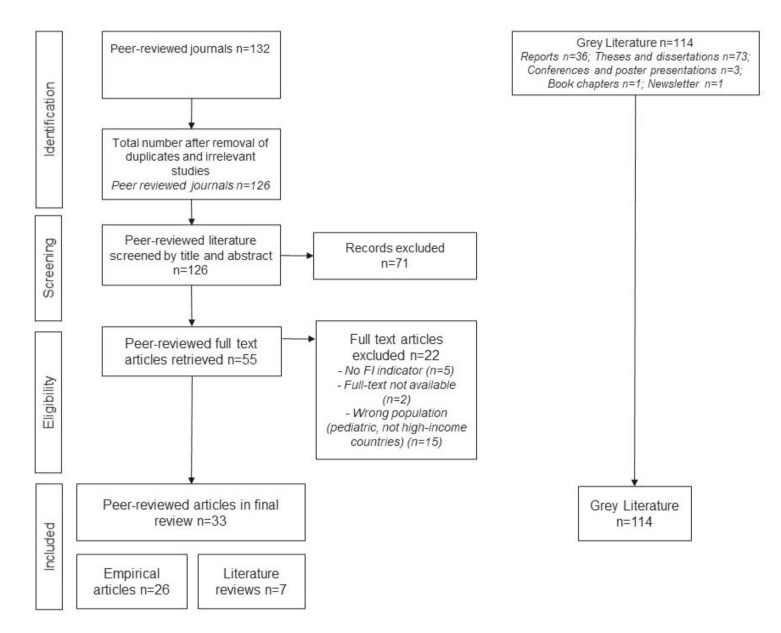
Flow diagram of study selection process. FI: Food insecurity.

For the screening and final selection of peer-reviewed journals, the software tool COVIDence was employed. This included uploading the peer-reviewed journals along with the full texts on COVIDence. Based on the suggestions of Arskey and O’Malley, the identification and selection of studies were a comprehensive and iterative process, which included weekly meetings in order to discuss the different phases and improve and direct the diverse aspects under agreement. Firstly, four research team members independently screened the titles and abstracts of the studies based on the eligibility criteria of this review. Based on a consensus, those documents which did not meet the eligibility criteria were eliminated from the study. Next, full texts of all potentially relevant documents were reviewed by the same four reviewers. Based on the consensus, few papers were eliminated. The process was reported following a flow chart from the extension for Scoping Reviews (PRISMA-ScR) [31], as shown in Figure 1.

### 2.3. Data Charting

Data were extracted, by pairs, by four members of the research team, and compared. A standardized form created by the research team was employed to input the data into Microsoft Excel Spreadsheets which was, based on the iterative process described above, continuously updated throughout the search strategy.

The data extracted for the peer-reviewed and grey literature was based on the following: title, author, year of publication, journal; country of development of the study; specific food insecurity indicator or indicators used; study population characteristics (age, country, household or individuals, respondents); procedure of data collection in the study (only in the peer-reviewed literature); results of the study.

### 2.4. Analysis of Results

The main findings were summarized using a narrative descriptive synthesis approach. A table containing relevant information about the different food insecurity measurement tools extracted from the selected peer-reviewed journals and grey literature of this review was completed, containing the name of the food insecurity instrument, its author/s and year; languages in which the instrument has been developed/translated; original purpose of development; unit to collect food insecurity data, i.e., individual or household; reference period of the assessment of food insecurity; dimensions of food insecurity; number of items; psychometric properties; types of response options and food insecurity classification; focus on quality and/or quantity of food intake; references in the review that have employed this particular instrument along with the country and level of food insecurity (individual-household); and strengths and limitations of this instrument. Data regarding the strengths and limitations of the instruments were retrieved from the documents using them as well as the documents reporting the creation and/or validation of their own measures.

## 3. Results

We initially identified 246 records—132 from the scientific literature and 114 from the grey literature. After removing duplicates and irrelevant studies from the scientific literature, 240 documents (126 from peer reviewed journals, 114 from the grey literature) were moved to the screening phase. Title and abstract screening eliminated 77 documents from the peer-reviewed literature. Afterwards, the remaining 55 records were assessed for eligibility using full text. Finally, 33 publications from the scientific literature were included (Figure 1), which comprised original articles (*n* = 26), literature reviews (*n* = 7), and the 114 documents from the grey literature.

Overall, these 147 documents reported data on food insecurity at the household level and individual level in high-income countries using a total of 23 measurement strategies.

In this section, we first describe the overall characteristics of the studies reported in these documents, separating the scientific and grey literature, to subsequently proceed to the depiction of the 23 measurements found in our sample.

### 3.1. Peer-Reviewed Literature

Table 1 shows the basic data of the peer-reviewed literature included in this scoping review. Over 90% of the reviewed empirical studies were conducted after 2015 (only [35,36] were published before that date). Eleven papers reported data on more than one country, with six of them including global samples of more than 100 countries [37,38,39,40,41,42], while the others used data from Europe [43], Macedonia, Moldova and Romania [44], and a combination of US and Canada [45]. The rest of the the papers used samples based in one country: US [35,46,47,48,49], Australia [50,51], Portugal [36,52], Canada [43,53,54], France [55], Greece [56], Italy [57], Norway [58] and Poland [59]. The remaining seven documents were literature reviews with no geographic restrictions, except for [60,61] which focused on combinations of US, Canada, England, New Zealand and Australia.

Three original papers explored the validity of new or existing food insecurity measures [37,39,51], while all others explored reported data on food insecurity prevalence with a different focus. Some studied its correlates: sociodemographics [40,58,59], age [52], health, social and poverty stressors [35] and self-confidence in resource management [47]; disparities based on gender [38,42,48], race [46] or region [36]; its health consequences [44,53,57,62]; and the impact of different policies and strategies [43,45,49,54,55,56,63]. The literature reviews’ objective was primarily to identify measurement tools available [16,21,22,23,60], although one of them also reviewed studies about food insecurity and environmental correlates [64], and another focused on gender disparities [61].

Fourteen original papers reported food insecurity at the household level [36,43,44,45,47,49,51,52,53,54,55,56,59,65] and twelve at the individual level, with only Park et al. [40] not focusing on the adult population (they collect data on the elderly). Literature reviews included both household and individual measures.

Instruments to assess household food insecurity included the 18-item Household Food Security Survey Module from USDA (18-HFSSM) [45,47,53,54,55,65], the Brazilian Food Insecurity Scale [36,52], the Household Food Insecurity Access Scale [59], the Food Security Survey Module (FSSM) [56], the Food Insecurity Index (FII) [49], the Household Hunger Scale (HHS) [44], the Healthy Diets ASAP [51] and the European Quality of Life Survey item “could your household afford a meal with meat, chicken or fish every second day if you wanted it?” [43]. Food Insecurity at the individual level was measured through the Food Insecurity Experience Scale (FIES), mostly through the Gallup World Survey [37,38,39,40,42,62], the six or ten-item Household Food Security Survey Module from USDA (6-HFSSM; 10-HFSSM) [35,46,48], the 10-item Radimer-Cornell Scale [58], the Household Food Insecurity Access Scale (HFIAS) [57] and an ad hoc developed single-item measure [50]. Other scales identified by the review papers include the Community Childhood Hunger Identification Project (CCHIP), the single-item National Health and Nutrition Examination Survey (NHANES-III), the USDA Food Insufficiency Question, the 10-item AFSSM, adapted HFSSM, the Australian National Health Survey single-item measure, the Household Food and Nutrition Security Survey (HFNSS), the USDA Food Insecurity and Hunger Scale, the New Zealand measure of individual deprivation, the Hager 2-item Food Insecurity Screening Questions, the Girard four point tool, the Kuyper past food insecurity screening, the Townsend Food Behaviour Checklist and other multi or single item measures.

Of the original studies using primary data (not previously existing surveys), the vast majority collected food insecurity information in face-to-face interviews. The study by Koh et al. [46] also uses online data collection. In those papers based on data from the GWP, food insecurity instruments are administered either face-to-face or via telephone. Nettle and Bateson’s [48] study also combines these two methods of data collection. Last, the procedure of data collection is not clearly stated for some studies, although is very likely that measurements have been taken in person [47,49,50,66,67].

**Table 1:** Overview of scientific literature of food insecurity studies: original articles and literature reviews.

Title	Author/s	Year	Journal	FI Measure	Country	Objective	Households/Individuals	Data Collection Procedure	Results
An Examination of Measurement Invariance Using a Multilevel Explanatory Rasch Model	Jue Wang, Victoria T. Tanaka, George Engelhard Jr. and Matthew P. Rabbitt [37]	2020	Measurement: Interdisciplinary Research and Perspectives	FIES	151 countries	To assess whether there is DIF in the FIES Scale due to gender and if so, to what extent. To study whether controlling for differences in the severity of latent food insecurity associated with person-level explanatory variables have an impact on the detection of gender DIF in the FIES Scale?	Individuals	Gallup survey (see note)	The results indicated the presence of differential item functioning for gender on the pooled (global) FIE Scale. Several person-level explanatory variables (educational attainment and socioeconomic status) also explained a significant amount of the variation in food insecurity measures. Furthermore, separate analyses were conducted for six geographic regions of the world, showing gender-related DIF, as well as the impact of person-level explanatory variables across the geographic regions
Confirmatory factor analysis to validate a new measure of food insecurity: Perceived and actual constructs	ElenaGrimaccia, Alessia Naccarato [39]	2020	Quality and Quantity	FIES	147 countries	To validate the FIES	Individuals	Gallup survey	FIES presents a good level of reliability and internal consistency. However, two distinct latent constructs were identified and analysed: A subscale measuring ‘perceived’ aspects of food insecurity and a subscale related to ‘actually experienced’ activities
Food Insecurity in Europe: A Gender Perspective	ElenaGrimaccia, Alessia Naccarato [38]	2020	Social Indicators Research	FIES	147 countries	To analyze the principal determinants of gender differences in food insecurity	Individuals	Gallup survey (see note)	The results suggest that the driver that could most mitigate women disadvantage is education: people with a university degree present a lower probability of experiencing food insecurity, both for men and for women. On the contrary, familial characteristics, such as the number of children in the household, present a higher impact on women’s food insecurity than men.
Food Insecurity among Small-Scale Farmersin Poland	Agnieszka Poczta-Wajda, Agnieszka Sapa, Sebastian Stepien, Michał Borychowski [59]	2020	Agriculture	HFIAS	Poland	To examine the level of food insecurity among small-scale farms in Poland	Household	Face-to-face interviews	The incidence and degree of food insecurity was measured with the Household Food Insecurity Access Scale (HFIAS). The study found that about 43% of the respondents were exposed to food insecurity, including almost 9% to severe food insecurity, which is well above the average for the entire Polish population. By applying cross-tabulation and the zero-inflated Poisson regression model, the study found that the higher age and secondary or higher education of the farm manager, having children in the household and higher land productivity have a statistically significant negative influence on households’ food insecurity (i.e., decreased HFIAS score). On the contrary, family size of five or more and production type “permanent crops” and “dairy cows” have a statistically significant positive influence on households’ food insecurity (i.e., increased HFIAS score).
Explaining racial inequality in food security in Columbus, Ohio: A blinder–oaxaca decomposition analysis	Keumseok Koh, Michelle L. Kaiser, Glennon Sweeney, Karima Samadi and Ayaz Hyder [46]	2020	International Journal of Environmental Research and Public Health	6-item HFSSM (USDA)	USA	Using Blinder-Oaxaca BO approach, this study aims to divide the Black–White food security differential into a part that is “explained” by group differences in socioeconomic characteristics, food shopping behaviors, and neighborhood perception and a remaining part that cannot be accounted for by such differences in the known determinants of food security in Columbus, Ohio.	Individuals (adults)	In-person or online survey	Compared with Black households, White households used their own cars more often and walk less to buy food, shopped 1.4 times more frequently, and travelled 0.6 miles less to acquire food in one month. Regarding neighborhood perception, White households were more satisfied with food accessibility in their neighborhood, and had more perceived connections with friends and neighbors in their communities. In terms of socioeconomic characteristics, White respondents were younger, possessed higher educational and household income levels, had fewer children in their homes, and were less likely to participate in SNAP. There was a 34.2 percent point (95% CI: 25.4–43.1) difference in food security between White and Black households.
Caregiver’s self-confidence in food resource management is associated with lower risk of household food insecurity among SNAP-Ed-eligible head start families	Lamis Jomaa, Muzi Na, Sally G. Eagleton, Marwa Diab-El-Harake and Jennifer S. Savage [47]	2020	Nutrients	18-item HFSSM (USDA)	USA	The present study aimed to first examine the associations between Food Resource Management (FRM) self-confidence and FRM behaviors by HFI status using a sample of SNAP-Ed-eligible Head Start families. A secondary objective of the study was to explore the association between financial practices of caregivers and HFI status in the study sample.	Households	N/A(most likely in person)	Participants with high FRM self-confidence had lower odds of HFI (OR = 0.54, 95%CI: 0.33, 0.87), yet FRM behaviors, financial practices, and HFI were not related after adjusting for covariates. All FRM self-confidence questions significantly differed by HFI, whereas only one of six FRM behaviors and two of three financial practices differed by HFI (all *p*-values < 0.05).
Association of maternal food insecurity before and during pregnancy with fetal structural anomalies: A multicenter case–control study protocol	Drieda Zac, Ilda Hoxhaj, TinaPasciuto, Rosario D’Anna, Gianluca Straface, Laura Reali, Marco De Santis and Maria Luisa Di Pietro [57]	2020	Nutrition and Health	HFIAS	Italy	The primary objective of the study was to investigate the impact of food insecurity among pregnant women before and during pregnancy on fetal structural anomalies Secondary objectives. Secondary objectives are to evaluate the prevalence of food insecurity among pregnant women in the study population, and to evaluate the prevalence of different types of fetal structural anomalies in the case group.	Individuals	N/A(most likely in person)	Finding a positive association between food insecurity in pregnant women and fetal structural anomalies could be the first step towards screening for it among pregnant women and designing policies that could mitigate this condition. Lowering food insecurity could prevent a certain number of fetal structural anomalies, leading to fewer negative pregnancy outcomes and health problems during childhood and adulthood
Hunger in Vulnerable Families in Southeastern Europe: Associations With Mental Health and Violence	Jansen, Elena; Lachman, Jamie M; Heinrichs, Nina; Hutchings, Judy; Baban, Adriana; Foran, Heather M [44]	2020	Frontiers in Public Health	Hunger Scale	North Macedonia, Republic of Moldova and Romania	This study explored the experience of hunger in vulnerable families in three South-eastern European countries, and simultaneously examined relationships with four sets of risk factors—lack of financial, mental, familial, and social resources	Household	Data collection took place either at participants’ homes, the study institutes or any agreed-on location.	Hunger in South-eastern European families, among families with children showing elevated behavioral problems, was associated with more family violence, but specifically poorer mental health and less emotional support above and beyond socio-structural strains. Adapting parenting interventions to support the primary caregiver in getting more access to emotional support may potentially also change hunger and its association with health and violence. However, this hypothetical pathway of change needs explicit testing
What explains gender differences in food insecurity?	Nzinga H. Broussard [42]	2020	Food Policy	FIES	2014 round of the GWP for 146 countries	To assess whether there are gender differences in FI	Individuals	Interviews are conducted either face-to-face or via telephone. Telephone surveys are conducted in countries with at least 80% telephone coverage.	In the developed countries of the European Union, women are 4.7 percentage points more likely than men to experience some form of food insecurity. In the poor countries of South Asia and Sub-Saharan Africa, women are two percentage points more likely than men to be severely food insecure
Heterogeneous factors predict food insecurity among the elderly in developed countries: Insights from a multi-national analysis of 48 countries	Jae Yeon Park, Arlette Saint Ville, Timothy Schwinghamer, Hugo Melgar-Quiñonez [68]	2019	Food Security	FIES	48 countries (GWP)	(1) to examine macro-level prevalence of food insecurity among the elderly in 48 developed countries. (2) to assess possible risk factors affecting food insecurity by the elderly in developed countries, using cross-nationally comparative methods.	Individuals (elderly)	Gallup survey	Food insecure individuals were more likely to live alone, not have a partner, and tended to have poorer scores for social support and wellbeing. Additionally, poor community infrastructure was associated with food insecurity of elderly people, and there were more food insecure elderly people in urban areas. At the multinational level, results indicated wide and statistically significant disparities among continents. The study concluded that not only personal factors but also social conditions could prevent the elderly from achieving full food security status.
“I worry if I will have food tomorrow”: A study on food insecurity among asylum seekers living in Norway	Sigrun Henjum, Marianne Sandsmark Morseth, Charles D. Arnold, Dawid Mauno, Laura Terragni [58]	2019	BMC Public Health	10-item Radimer/Cornell Hunger and Food Insecurity Scale	Norway	to assess food security among asylum seekers living in Norwegian reception centers	individuals	Face-to-face	Seven percent of the participants were categorized as food secure and 93% as food insecure, of whom 11% were food insecure without hunger, 78% were food insecure with hunger, and 4% were food insecure with child hunger. Among the families with children, 20% (8 of 41) experienced child hunger. For the participants experiencing food insecurity with hunger, 44% reported that they were hungry often, and among families with children, 14% reported that despite being aware of the child’s hunger, they did not have the resources/money to buy more food. In logistic regression models, men had higher odds of experiencing adult food insecurity with hunger than women, OR (95% CI): 4.08 (2.04, 8.16). A reduction in monthly budget by 100 euros increased the odds of experiencing adult food in-security with hunger by 1.37 times OR (95% CI), 1.37 (1.16, 1.61)
Food-Insecure Women Eat a Less Diverse Diet in a More Temporally Variable Way: Evidence from the US National Health and Nutrition Examination Survey, 2013-4	Daniel Nettle and Melissa Bateson [48]	2019	Journal of Obesity	10-item HFSSM	USA	we investigated in detail the 24-h. food-consumption recalls of adult women in the 2013-4 cycle of NHANES. Like previous studies, we extracted variables concerning total energy intake, macronutrient composition, and number of eating occasions in the day.	Individuals	two separate food recall interviews, the first in person and the second by telephone	Compared to the food-secure, food-insecure women had more variable time gaps between eating; ate a smaller and less variable number of distinct foods at a time; were more variable from day to day in their time of first consumption; were more variable from day to day in the number of times they ate; and consumed relatively more carbohydrate, less protein, and less fibre. However, their overall energy intake was no higher. Food insecure women had higher BMIs (2.25 kg/m^2^), and around 15% of the BMI difference between food-insecure and food-secure women was accounted for by their more variable time gaps between eating, their lower diversity of foods, and their lower fibre consumption.
Examining the Association between Food Literacy and Food Insecurity	Andrea Begley; Ellen Paynter; Lucy M. Butcher; Satvinder S. Dhaliwal [50]	2019	Nutrients	Ad hoc single item FI measure ENEP	Australia	The aim of this research was to describe the apparent prevalence of food insecurity in adults at enrolment in a food literacy program and to examine the relationship between food insecurity and a range of independent variables.	Individuals	Individuals were encouraged where possible to complete a questionnaire before starting the first session	The results are salient as they indicate an association between food literacy and food insecurity. The implications are that food insecure participants may respond differently to food literacy programs. It may be necessary to screen people enrolling in programs, tailor program content, and include comprehensive measures in evaluation to determine effect on the impact of food literacy programs on different subgroups
Money speaks: Reductions in severe food insecurity follow the Canada Child Benefit	Brown, E.M.; Tarasuk, V. [65]	2019	Preventive Medicine	18-item HFSSM	Canada	To assess whether Canadian households with children experienced reductions in food insecurity compared to those without following the roll-out of a new country-wide income transfer program: the Canada Child Benefit (CCB).	Households (adults and children)	NA	Multinomial logistic regressions were used to test the association between CCB and food insecurity among three samples: households reporting any income (*n* = 41,455), the median income or less (*n* = 18,191) and the Low-Income Measure (LIM) or less (*n* = 7579). The prevalence and severity of food insecurity increased with economic vulnerability, and were both consistently higher among households with children. However, they also experienced significantly greater drops in the likelihood of experiencing severe food insecurity following CCB; most dramatically among those reporting the LIM or less (DID: −4.7%, 95% CI: −8.6, −0.7). These results suggest that CCB disproportionately benefited families most susceptible to food insecurity. Furthermore, our findings also indicate that food insecurity may be impacted by even modest changes to economic circumstance, speaking to the potential of income transfers to help people meet their basic needs.
Impact of fruits and vegetables vouchers on food insecurity in disadvantaged families from a Paris suburb	Buscail, C.; Gendreau, J.; Daval, P.; Lombrail, P.; Hercberg, S.; Latino-Martel, P.; Julia, C. [55]	2019	BMC Nutrition	18-item HFSSM	France	To assess the impact of fruits and vegetables vouchers on food security among disadvantaged households from a Paris suburb.	Households	Face-to-face at community centers or at home	Among the 91 families included between May 2015 and May 2016, 64 completed the post assessment questionnaire. At inclusion, 68.3% of families were experiencing food insecurity and 78.1% were experiencing food insufficiency. No association was found between food consumptions and food security status. After one-year follow-up, the prevalence of food insufficiency was significantly decreased in the intervention group (61.8%, with *p* value = 0.03), and unchanged in the control group. Conclusion: In this pilot study, food insufficiency was significantly decreased in families receiving vouchers for fruits and vegetables over a one-year period
Food Insecurity Is More Strongly Associated with Poor Subjective Well-Being in More-Developed Countries than in Less-Developed Countries	Frongillo, E.A. Edward A.; Nguyen, H.T. Hoa T.; Smith, M.D. Michael D. M.D. Michael D; Coleman-Jensen, Alisha [62]	2019	Journal of Nutrition	FIES	Data from the Gallup World Poll 2014 in 147 countries	We aimed to deepen understanding of the relation between food insecurity and subjective well-being among countries from the perspective of possible hedonic adaptation between food insecurity and subjective well-being	Individuals	Gallup survey	The prevalence of food insecurity was strongly and negatively associated with subjective well-being across 147 countries. The association between food insecurity and poor subjective well-being within countries was stronger for more-developed countries, providing evidence of hedonic adaptation between food insecurity and subjective well-being. Food insecurity explained substantial variation in subjective well-being both among and within countries
Food insecurity status and mortality among adults in Ontario, Canada	Craig Gundersen, Valerie Tarasuk, Joyce Cheng, Claire de Oliveira, Paul Kurdyak [53]	2018	Plos One	18-item HFSSM	Canada	to ascertain the association between food insecurity and all-cause mortality for a population-based sample of adults.	Household	NA	Using a full set of covariates, in comparison to food secure individuals, the odds of death at any point after the interview are 1.28 (CI = 1.08, 1.52) for marginally food insecure individuals, 1.49 (CI = 1.29, 1.73) for moderately food insecure individuals, and 2.60 (CI = 2.17, 3.12) for severely food insecure individuals. When mortality within four years of the interview is considered, the odds are, respectively, 1.19 (CI = 0.95, 1.50), 1.65 (CI = 1.37, 1.98), and 2.31 (CI = 1.81, 2.93)
Testing the price of healthy and current diets in remote aboriginal communities to improve food security: Development of the aboriginal and torres strait islander healthy diets ASAP (Australian standardised affordability and pricing) methods	Amanda Lee and Meron Lewis [51]	2018	International Journal of Environmental Research and Public Health	Healthy Diets ASAP	Australia	The aim of this study was to modify and test the Healthy Diets ASAP methods protocol to be more relevant to the Aboriginal and Torres Strait Islander population. It developed methods and tools to assist others to apply the approach in order to compare the price, price differential and affordability of healthy (recommended) and current (unhealthy) diets of Aboriginal and Torres Strait Islanders living in different locations with other population groups in Australia.	Household	Face-to-face interviews	
The rise of food banks and the challenge of matching food assistance with potential need: Towards a spatially specific, rapid assessment approach.	Christopher M. Bacon and Gregory A. Baker [49]	2017	Agriculture and Human Values	Food Insecurity Index (FII)	USA	The objective of this study is to assess how well a local food assistance organization serves its clientele from a geo- graphical perspective. Specifically, the research question is: do the areas with the highest concentration of people who are likely in need of food assistance have food distribution sites in close proximity?	Households	Household data from US Census ACS 5-year estimates of poverty and demographic data and data from a local food bank	The findings suggest that food assistance distribution locations match the areas of potential need in more than 80% of urban census tracts. However, there are several potentially underserved locations and populations that could benefit from new food assistance operations.
Did food insecurity rise across Europe after the 2008 crisis? An analysis across welfare regimes	Davis, Owen; Geiger, Ben Baumberg [43]	2017	Social Policy and Society	European Quality of Life Survey item ‘could your household afford a meal with meat, chicken or fish every second day if you wanted it?’	Europe	First, we explore whether food insecurity has risen since the 2008 crisis as the rise in food aid suggests. Second, we examine if this rise has varied across welfare regimes, if it has occurred at all.	Household	interviews were conducted face-to-face in respondents’ own homes using standardised question wording.	The article finds evidence to support both contentions: food insecurity has risen across many European countries and has varied by welfare regime. It also finds that contrary to expectations, the sharpest rise was in the Anglo-Saxon countries of Ireland and the UK, rather than Southern or Eastern European countries.
The impact of changes in social policies on household food insecurity in British Columbia, 2005–2012	NaLi, Naomi Dachner, Valerie Tarasuk [54]	2016	Preventive Medicine	18-item HFSSM	Canada	The primary objectives of this study were to describe the socio- demographic and temporal patterning of food insecurity in BC from 2005 to 2012 and determine whether BC’s increase in social assistance and introduction of the RAP affected food insecurity among the target groups. A secondary objective was to compare the sensitivity of different levels of household food insecurity to these two policy interventions.	Households	NA	Overall food insecurity rose significantly among households in BC between 2005 and 2012. Following the increase in social assistance benefits, overall food insecurity and moderate and severe food insecurity declined among households on social assistance, but severe food insecurity remained unchanged.
The impact of a school food aid program on household food insecurity	Athanassios Petralias; Eleni Papadimitriou; EleN/ARiza; Margaret R. Karagas; Alexia B.A. Zagouras; AthenaLinos [56]	2016	European Journal of Public Health	Food Security Survey Module (FSSM)	Greece	We hypothesized that children and their families living in low SES districts experience food insecurity and that the food aid program would reduce its rates. This is the first program of this type and magnitude in Greece	Household	NA	59.1% post-intervention, *p* < 0.001. On an individual level, food insecurity score diminished by 6.5%, *p* < 0.001. After adjustment for various socioeconomic factors, for each additional month of participation, the odds of reducing the food insecurity score increased by 6.3% (OR = 1.06, 95% CI: 1.02–1.11). Those experiencing food insecurity with hunger at baseline were more likely to improve food insecurity score than those who did not (OR = 3.51, 95%CI: 2.92–4.21).
Understanding Food Insecurity in the USA and Canada: Potential Insights for Europe	Gundersen, C. [45]	2016	World Rev Nutr Diet.	18-item HFSSM	US/Canada	Given the similarities between the USA, Canada, and Europe, previous research can offer numerous insights into the causes and consequences of food insecurity in Europe and possible directions to address these through measurement and public policies.	Households	NA	It first covers the methods used to measure food insecurity in the USA and Canada. In both countries, a series of 18 questions in the Core Food Security Module are used to identify whether a household is food insecure. It then briefly covers the current extent of food insecurity in each country along with some discussion of the recent history of food insecurity. A central advantage to using the Core Food Security Module in Europe is that the measure has been proven useful in other high-income countries, and using a standardized measure would allow for cross-country comparisons. I next cover two large-scale food assistance programs from the USA, the Supplemental Nutrition Assistance Program (formerly known as the Food Stamp Program) and the National School Lunch Program. For each, I summarize how the program is structured, how eligibility is established, and how participation proceeds. Europe has generally used income-based assistance programs to improve the well-being of low-income households; I consider a couple of reasons for why food assistance programs may also be worth considering.
Time and regional perspectives of food insecurity during the economic crisis in Portugal, 2011–2013	Graca, P.; Gregorio, M.J.; Costa, A.; Nogueira, P.J. [36]	2014	Saúde Soc. São Paulo,	FI was assessed using a psychometric scale adapted from the Brazilian Food Insecurity Scale (IBGE, 2010)	Portugal	This study aims to evaluate trends in FI prevalence during the economic crisis in Portugal and to identify regional disparities throughout the country	Household	Data were collected using face-to-face interviews by nurses in primary health care	The prevalence of FI was relatively unchanged at national and regional levels, during the analysis period. Data from 2013 indicates a high prevalence of FI (50.7%), including 33.4% for low FI, 10.1% for moderate FI and 7.2% for severe FI. Disparities according health region were also found for household FI. Algarve, Lisbon and Vale do Tejo were the two regions with the highest levels of FI, even after controlling or other socioeconomic variables. High levels of FI found in Portugal and the different regional profiles suggest the need for regional strategies, in particular in the most affected regions based on a broader action with different policy sectors (health, social security, municipalities and local institutions in the field of social economy).
Stress and Poverty Predictors of Treatment Adherence Among People With Low-Literacy Living With HIV/AIDS	Kalichman, Seth C and Grebler, Tamar [35]	2010	Psychosomatic Medicine	6-item HFSSM	USA	To examine the association of social, health, and poverty-related stressors in relation to antiretroviral therapy adherence in a sample of people with low-literacy living with HIV/AIDS in the South-Eastern United States. Emotional distress is among the more common factors associated with HIV treatment adherence.	Individuals	Face-to-face interviews	Two-thirds of the sample demonstrated adherence <85% of pills taken. Multivariable analyses showed that food insufficiency and hunger predicted antiretroviral therapy nonadherence over and above depression, internalized stigma, substance use, and HIV-related social stressors
Food insecurity and aging during economic crisis	Graça, P., Gregoório, M.J. [52]	2017	Psychology and Behavior	Brazilian Food Insecurity Scale	Portugal	To assess food insecurity prevalence across age groups in Portugal.	Households	Face-to-face interviews	This national survey on food insecurity, implemented in 2011 in order to evaluate food insecurity trends during the period that Portugal was under the International Monetary Fund financial assistance program (2011–2014), found a high prevalence of food in- security in the Portuguese population. In 2014, 45.8% of the Portuguese households included in this study were food insecure. A higher risk of food insecurity was observed in the age groups of 30–64 years, when comparing to adults aged 65 and over. Data from Infofamiília survey in Portugal are consistent with results obtained in other countries.
Food insecurity and mental health among females in high-income countries	Merryn Maynard, Lesley Andrade, Sara Packull-McCormick, Christopher M. Perlman, Cesar Leos-Toro and Sharon, I. Kirkpatrick [61]	2018	International Journal of Environmental Research and Public Health	18-item HFSSM (USDA); single-item from Radimer Cornell; Community Childhood Hunger Identification Project (CCHIP); single-item National Health and Nutrition Examination Survey (NHANES-III); New Zealand measure of individual deprivation, Other multi or single item measures	USA, Canada, New Zealand and England	to examine the state of the literature on food insecurity and mental health among women living in high-income countries	Individuals (women)	N/A	Most research was cross-sectional and showed associations between depression and food insecurity; longitudinal analyses suggested bidirectional relationships (with food insecurity increasing the risk of depressive symptoms or diagnosis, or depression predicting food insecurity). Several articles focused on vulnerable subgroups, such as pregnant women and mothers, women at risk of homelessness, refugees, and those who had been exposed to violence or substance abuse. Overall, this review supports a link between food insecurity and mental health (and other factors, such as housing circumstances and exposure to violence) among women in high-income countries and underscores the need for comprehensive policies and programs that recognize complex links among public health challenges.
Place and food insecurity: A critical review and synthesis of the literature	Megan Ann Carter, Lise Dubois and Mark S Tremblay [64]	2014	Public Health Nutrition	18-item HFSSM; 6-item HFSSM; Community Childhood Hunger Identification Project; Radimer Cornell measure; adapted version of Radimer Cornell measure	N/A(systematic review)	The present review sought to synthesize and critically appraise the existing literature examining local environmental characteristics in relation to individual/household-level food insecurity in the general population.	Households and individuals	This review chose those research studies for inclusion if they examined the relationship between features of place and self-reported food insecurity (either at the household/family or individual level).	After obtaining full-text articles, eighteen primary studies met the eligibility criteria. Most studies were conducted in the USA and all but one was cross- sectional. Seven of the eleven studies that examined location of residence found that rural living was inversely associated with food insecurity. Mixed results were seen for other place measures such as social capital and distance to food stores. Conclusions: Studies were heterogeneous and had various limitations that preclude definitive conclusions from being drawn. Recommendations for future research are provided.
Investigating food insecurity measurement globally to inform practice locally: A rapid evidence review	Emma Beacom, Sineéad Furey, Lynsey Hollywood and Paul Humphreys [22]	2020	Critical Reviews in Food Science and Nutrition	18-item HFSSM; 6-item HFSSM; FIES; USDA Food Insufficiency Question; 10-item AFSSM; adapted HFSSM; Australian National Health Survey single-item measure; Household Food and Nutrition Security Survey (HFNSS); USDA Food Insecurity and Hunger Scale	N/A(systematic review)	The objective of this review was to examine the methods used to measure food insecurity (FI) globally, to inform considerations relating to adopting a novel, or reviewing an existing, FI measurement approach in developed countries.	Households and individuals	Focus groups, surveys, secondary data analysis, and interviews	Results found that the majority of papers reviewed emanate from North America with the US Household Food Security Scale Module (HFSSM) and its various adapted forms being the most commonly reported indicator. FI is becoming a key concern within developed countries with a range of indicators being used to report on the severity of the issue.
Identifying a potential tool to measure household food insecurity in the UK: A systematic review	G. Nguyen, L. Aucott, G. McNeill, F. Douglas [21]	2017	Proceedings of the Nutrition Society	Twenty different tools were identified with the most commonly used being the USDA Household Food Security Survey Module (HFSSM) (66%), the Canadian HFSSM (8%). Other tools such as the Radimer/Cornell Hunger Scale, FAO Food Insecurity Experience Scale (FIES), etc. had only been used in a small proportion of the included studies	N/A(systematic review)	to identify the measurement instruments which have been used to measure HFI in developed/high income countries which would be suitable for use in the UK context.	Households and individuals	NA	There are a number of structured scales available in the literature for measuring HFI, the USDA HFSSM stands out as the most commonly used tool in high income countries. Additional work to develop questions to better capture the psychological and social components of HFI is required.
Measuring food insecurity	Castetbon, K. [16]	2016	Book chapter	Radimer/Cornell Cornell Child Food Security Measure Community Childhood Hunger Identification Project (CCHIP) tool Food Security Core Module (FSCM), also named US HFSS	N/A(systematic review)	reviews studies into food security at the level of the household and of the individual, focusing especially on the question of how to define and measure the complex concept of food insecurity.	Households and individuals	NA	The concept of ‘food security’ has been under discussion since the 1970s, and an extensive literature on it has existed since the early 1990s. • Research into food insecurity has focused primarily on the developing world, but food insecurity also exists in the developed world. Questionnaires designed to measure food insecurity most commonly focus on the level of the individual and of the household. • Questionnaires designed to measure food insecurity have become progressively more sophisticated. • The definition of ‘food insecurity’ is still in flux. • Existing data on food insecurity are too diverse to admit a simple synthesis, but further research will generate findings that will permit a thoroughgoing analysis of the phenomenon.
Measurement of household food security in the USA and other industrialised countries	Kathy L. Radimer [60]	2002	Public Health Nutrition	18-item HFSSM (USDA); 6-item HFSSM; USDA Food Insufficiency Indicator; 8-item Food Security Survey (Reid, New Zealand); 2-item adapted from Radimer Cornell (Australia); National Population Health Survey (Canada)	USA, New Zealand, Australia, Canada	To describe the history and current status of household food security measurement in the USA and other industrialised countries.	Households and individuals	N/A	Current research on food security measurement includes measurement of individual food insecurity and hunger, module performance regarding hunger duration and frequency, performance of the module in population sub- groups, and the effect of translations on module meaning and performance. National surveys in Canada, New Zealand and Australia also have measured food security.
Measurement of the dimensions of food insecurity in developed countries: A systematic literature review	Ashby, Stephanie; Kleve, Suzanne; McKechnie, Rebecca; Palermo, Claire [23]	2016	Public Health Nutrition	Radimer/Cornell Cornell Child Food Security Measure Community Childhood Hunger Identification Project (CCHIP) tool Hager two-item screen Girard four-point tool Kuyper part food insecurity Household Food Insecurity Access Scal (HFIAS) Townsend Food Behavior Checklist	N/A(systematic review)	The aim of the present study was to conduct a systematic literature review to identify all multi-item tools that measure food insecurity and explore which of the dimensions they assess.	Households and individuals	NA	Eight multi-item tools were identified. All of the tools assessed the ‘food access’ dimension and two partially assessed the dimensions ‘food utilization’ and ‘stability over time’, respectively. ‘Food availability’ was not assessed by existing tools. Conclusions: Current tools available for measuring food insecurity are subjective, limited in scope, with a majority assessing only one dimension of food insecurity (access). To more accurately assess the true burden of food insecurity, tools should be adapted or developed to assess all four dimensions of food insecurity

Note. Galluop surveys: Gallup uses telephone surveys in countries where telephone coverage represents at least 80% of the population or is the customary survey methodology. In the developing world, including much of Latin America, the former Soviet Union countries, nearly all of Asia, the Middle East, and Africa, Gallup uses an area frame design for face-to-face interviewing in randomly selected households.

### 3.2. Grey Literature

The grey literature part of the sample is composed by the thesis (*n* = 73), research and institutional reports (*n* = 36), conferences (*n* = 3), book chapters (*n* = 1) and newsletter pieces (*n* = 1). For periodical reports (like the FAO’s SOFI), we have only considered the last version. Twenty records were published between 2000 and 2010, but the vast majority (*n* = 76) are posterior to 2015. 

With regard to the countries where the data was collected, three of them report data from several countries—some including only developed but most also developing countries [2,68,69] five of them come from Australia [70,71,72,73,74], one from New Zealand [75], five from the UK [76,77,78,79,80], two from Spain [81,82], and 24 from Canada [83,84,85,86,87,88,89,90,91,92,93,94,95,96,97,98,99,100,101,102,103,104]. The remaining 73 documents report data from the US [105,106,107,108,109,110,111,112,113,114,115,116,117,118,119,120,121,122,123,124,125,126,127,128,129,130,131,132,133,134,135,136,137,138,139,140,141,142,143,144,145,146,147,148,149,150,151,152,153,154,155,156,157,158,159,160,161,162,163,164,165,166,167,168,169,170,171,172,173].

Most documents provide data on the prevalence and correlations of food insecurity in the different territories and population groups, although a small sample (*n* = 4) also delves into the effect of interventions [99,141] or policy options [65,94].

Food insecurity was measured at the household level in 49 of the records, being the most used instruments the 18-item HFFSSM (*n* = 39), the 10-item HFSSM (*n* = 4), the 6-item HFSSM (*n* = 3), the single-item food insecurity question from the Australian NSW Population Health Survey (*n* = 1), the FIES scale (*n* = 2), the European Quality of Life Survey item “could your household afford a meal with meat, chicken or fish every second day if you wanted it?” (*n* = 1); Current Population Survey Food Security Supplement (CPS FSS), USDA (*n* = 1). Note that some of the studies use more than one measurement instrument.

The remaining 67 documents assessed food insecurity at the individual level, with a variety of profiles: three focused on adolescents, and used the 9-item CFSSM (*n* = 2) and the single-item Food Insecurity Measure (*n* = 1). Most records measured food insecurity in adults (*n* = 31), four of them being exclusively on women and used the 6-item and 18-item HFSSM, the 2005 Oregon PRAMS single-item Food Insecurity Measure and the HFIAS Scale. The other studies on adults comprise both gender and measure food insecurity through the 6-item (*n* = 10), 10 item (*n* = 6), 18-item HFSSM (*n* = 5), the Radimer-Cornell Food Insecurity Measure (*n* = 1), the 8-item food security questionnaire by Reid (1997) (*n* = 1), the single item Food Insecurity measure (*n* = 1), the Food Security Module at the LISA Study (*n* = 1) and the HFIAS scale (*n* = 1). Five additional studies were conducted on adults and children, and used the FIES scale (*n* = 2), 6-item HFSSM (*n* = 1) and 18-item HFSSM (*n* = 2); 23 on College students, and measured food insecurity through the 6-item HFSSM (*n* = 14), 10 item HFSSM (*n* = 2) and 18-item HFSSM (*n* = 7); and four documents reported measures of food insecurity on elderly, using the 6-item HFSSM (*n* = 2) and 10-item HFSSM (*n* = 1) and FIES scale (*n* = 1) (see Table 2).

### 3.3. Measures of Food Insecurity

Overall, our literature review identified 23 measurement tools to assess food insecurity at the individual or household level in high-income countries (Table 3). The most used instruments are those of the USDA HFSSM which together (6, 10, 18 item versions) have been used in 71.8% percent of our sample. These are followed by the FIES (8%), HFIAS (2.7%) and the Single-item Food Insecurity question from the Australian NSW Population Health Survey (2%). The remaining 17 instruments were each used by one or two documents in our sample.

The majority of instruments identified in this study were developed by institutions with the purpose of monitoring food insecurity, its severity and its correlations in several contexts. The first tools (Radimer-Cornell FIM; 18-item, 10-item and 6-item HFSSM), date from the nineties, whereas more than half of the instruments have been developed since 2005.

In total, 74% of instruments in our sample were validated. Not validated measures or measures for which validation information has not been found include the Australian single-item, the 2005 Oregon PRAMS single-item, the ENEP single-item, the question in the European Quality of Life Survey, the Food Insecurity Index, and the Healthy Diets ASAP. We found that 65.2% of the existent instruments in our sample measure food insecurity at the household level, while 17.4% measure food insecurity at the individual level (10-item HFSSM/AFSSM, CFSSM, Townsend Food Behaviour Checklist, and ENEP single-item) and 17.4% at both the household and individual level (Girard four point tool, LISA Food Security Module, Australian single-item, 6-item HFSSM). The target population of the most commonly employed food insecurity instruments such as the US-HFSSM (18-item and 6-item) as well as the HFIAS and FIES include all age groups within the household, i.e., children, adults and the elderly. The CFSSM of the USDA is the only validated child food insecurity instrument for children and adolescents aged 12 years or above. There are some differences between the US-FSSM and CFSSM. The CFSSM has been developed from the HFSSM; however, the former is simple without any screeners, easier to understand and can be self-administered in older children and teenagers. The keywords in each of the nine items of the CFSSM (i.e., worry, run out, cheap food, etc.) are underlined in order to ensure that the child comprehends the question.

Regarding the dimensions of food insecurity captured by the different instruments in our sample, it was evident that all the instruments in our sample focus on accessibility and availability of food mostly related to financial constraints. The USDA’s instruments specifically focus on the psychological aspects of food insecurity concerning worry or stress and anxiety over not being able to procure or consume sufficient food. On the other hand, the eight-item New Zealand Food Insecurity measurement tool along with focussing on the psychological aspects of food insecurity also question participants about food assistance from family or other sources and reliance on food banks. It is evident from the instruments in our sample that the majority of them (83%) mainly focus on the quantity of food. Only 17% of the instruments in our sample, which include the Radimer Cornell Food Insecurity Measure, Brazilian Food Insecurity Scale, HFNSS and Healthy Diets ASAP, capture both the quantitative and qualitative aspects of food insecurity. Further, the Brazilian Food Insecurity Scale focusses on additional and interesting aspects of food insecurity including family expenses and food consumption while the Healthy Diets ASAP also covers the pricing of healthy food and drinks.

All the instruments in our sample have been developed in English including the Brazilian Food Insecurity Scale which was developed in Portuguese but includes an English version. 73.8% of instruments in our sample belong to the USDA which has developed different versions of its original HFSSM, including shorter versions and the self-reported module for older children or adolescents. The instruments in our sample have been most commonly used in the US (61%).

As noted in Table 4, some of the instruments have a large number of items in order to capture the perceptions of food insecurity in participants more effectively. The HFNSS has 26 items in its instrument while the US-HFSSM and LISA Food Security Module have 18 items. On the other hand, there are some instruments only use a single item in order to measure food insecurity such as the NSW Population Health Survey, single item Australia, Oregon PRAMS, ENEP and EQLS. These instruments are simple and easy to employ yet may not be able to capture all aspects of food insecurity.

Most of the food insecurity instruments in our sample including all of the USDA’s instruments have a time frame of either 30 days or 12 months. The psychometric properties of all validated instruments (74%) in our sample have been found to be good. As noted in Table 4, we have identified several reported strengths of the different food insecurity instruments especially the US-HFSSM, which is considered to be a comprehensive survey that captures different levels of severity of food insecurity within the household. At the same time, various issues and unmet challenges have been described regarding these tools, which need to be addressed. The underlying issue that arises with most of these instruments including the US-HFSSM is that it does not capture the qualitative aspects of food insecurity. However, some of the challenges that have been found in these instruments are related to respondent burden due to the lengthy nature of the survey as in the case of the 18-item US-HFSSM, whereas shorter versions, like the Australian single-item FI indicator, may not adequately capture the intensity and complexity of food insecurity. Notwithstanding that, the main issue as in the case of the commonly used food insecurity indicators such as the US-HFSSM is related to the lack of items questioning the respondent about dietary quality.

## 4. Discussion

Research on food insecurity in high-income countries is only increasing—as observed by the growing number of papers and other documents published in recent years. This trend actually reflects a highly noticeable social and health-related issue, which is the alarming volume of households and individuals in the rich world that cannot see their fundamental right to adequate food guaranteed, as recognized by the Universal Declaration of Human Rights [180]. In a more recent version, the Sustainable Development Goals (especially SDG 2 and 3) emphasize the relevance of tackling this situation—and in fact, the FIES Scale was developed as a tool to assess the progression of SDG2 [181]. It is also urgent to adequately assess and monitor the scope of food insecurity, as it is by better comprehending the extent, correlations, causes and consequences of such phenomena in the different contexts that it will be possible to provide the most adequate solutions. One of the biggest challenges to this endeavor is the complexity of the phenomenon of food insecurity itself, which, when considering all its dimensions (availability, access, use and stability), encompasses not only the entirety of the food system, but also the personal and household experiences of those suffering from it. In this way, it adds a layer of intricacy, as it mixes objective and subjective perspectives.

In our review, we have identified 23 instruments to assess food insecurity at the individual or household level in high-income countries. The most used instruments are those of the USDA HFSSM followed by the FIES, HFIAS and the Single-item Food Insecurity question from the Australian NSW Population Health Survey [2,67,68,175]. These scales assess the experience of food insecurity at the access dimension, either at the household or individual level. Assessing the experience of food insecurity means that it is the subjective perception of the respondents about food what is being considered (i.e., in the questions such as the HFIAS In the past four weeks, were you or any household member not able to eat the kinds of foods you preferred because of a lack of resources? or In the past four weeks, did you or any household member have to eat a limited variety of foods due to a lack of resources?). It is interesting to note that although some of the studies identified in this review focused on specific populations like women, college students or the elderly, the scales used are not unique for this target—something that in the case of the elderly might be considered, given their particular conditions [25].

The fact that the USDA HFSSM scales have been the most used in our sample (in more than 70% of the documents), is coherent with the point that we are studying food insecurity in high-income countries [2]. The FIES and HFIAS were initially conceived to be used in low-income countries, but they have also been used in high-income ones, either for comparative purposes [2,69] or for selected deprived areas or populations [59,182]. In any case, the three sets of scales share a high number of common items, since the HFIAS was developed based on the HFSSM scales and has, in turn, informed the development of the FIES scale together with the Latin American and Caribbean Food Security Scale (ELCSA) [25,183]. They differ from each other by including or not including aspects related to the possibility of satisfying one’s (household) food preferences (HFIAS) or accessibility to nutritious food (HFSSM/FIES). In this sense, they collect information that refers to the contextualization aspects of the concept of food insecurity [17] since these seem to be elements more relevant to the subjective experience of food security in high-income societies—where availability of a wide variety of foods is guaranteed.

Since dietary intake is one of the main determinants of health [184], food insecurity must be considered a relevant axis of health inequalities, and dietary quality should be considered in any action developed to reduce it. Therefore, we agree with Shanks [185] that the measurement of dietary diversity (and we may add food quality) to characterize food insecurity is essential. This is in fact one of the missing points of current scales to measure food insecurity—in our review, only three of the instruments found assessed both quantity and quality aspects of food intake, and were the least used. Moreover, although there exist specific instruments to measure it (such as the Household Dietary Diversity Score), these instruments are yet not very much implemented. In that sense, special interest was observed among the instruments used in the literature to add items considering health and socioeconomic aspects related with food insecurity such as family income, expenses and food consumption, but also structural issues like social programs and policies, which should systematically be included in food insecurity studies.

Assessing dietary diversity entails a challenge that echoes some of the traditional disputes on the measurement of food insecurity [28], namely the use of objective or subjective/experiential scales. In this way, while being able to capture people’s (and their household’s) experiences of food insecurity is essential, characterizing adequate food intake requires using objective measures. It means that while questions like the FIES “You were unable to eat healthy and nutritious food?” or the HFSSM “(I/we) couldn’t afford to eat balanced meals” provide information about the respondent’s perceptions of their food access possibilities, it might not fully reflect what from a public health perspective would be considered a proper diet. Additionally, given how current food systems have evolved with the proliferation and accessibility of ultraprocessed foods, looking only at the number of food groups consumed may not suffice to gain a proper view of diet quality, and therefore it might be necessary to complement current food insecurity scales with other traditional food intake recalls such as food diaries, 24 or 72 h recalls or food frequency questionnaires. In addition, another important aspect to take into account is that access to drinking water (water security) is not valued.

The scales identified in this review focus on the access dimension of food insecurity, and efforts should be made to also capture the use dimension, as well as other relevant aspects such as the environment of adequate sanitation, health services and care, allowing for a healthy and active life which the FAO 2012’s definition of food insecurity recognizes [14]. This is something that Pereira and colleagues [186] described in the case of children, and is applicable to the case of adults as well.

Of course, reaching such a rich amount of data on the different dimensions of food security and its determinants (and consequences) may be at odds with the practicalities of data collection. It is in this case that the range of options available—in terms of number of items and length of the scales—must be considered to obtain the more complete data, as compatible with the requirements and possibility of each study.

This review is not exempt from limitations. Mainly, the algorithm used to identify the literature uses the term “high-income countries”, but no individual country name. In this way, some single-country studies may have not been identified. However, the inclusion of gray literature and the considerably sizeable sample make it a complete reference to provide a comprehensive view of the instruments used to measure individual and household food insecurity in high-income countries.

## 5. Conclusions

This work supplements and expands on previous reviews on measures of food insecurity in high-income countries by maximizing the searched databases, applying no limits to the nature of the measure (single-item, survey-based, etc.) and including scales used in studies published in the gray literature. In this way, it offers an updated and comprehensive catalogue of instruments to measure food insecurity at the individual and household level in high-income to be used by both researchers and practitioners.

As it has been previously recognized, no ‘perfect single measure that captures all aspects of food insecurity’ exists, but efforts should continue to improve our understanding of food insecurity, combining both objective and subjective elements of assessment. Therefore, this scoping review considered that further research is required in terms of (i) the creation and/or improvement of validated measurement for food insecurity in high-income countries, (ii) the incorporation of food quality and dietary intake items to assess the food use, and (iii) the consideration of aspects such as the household composition, the economic situation of the individuals and households, their use of social resources and food aid and their food literacy and competences.

## Figures and Tables

**Table 2:** Overview of Grey Literature in food insecurity studies, theses, reports, conferences and newsletters.

Type of Document	Author/s	Title (Year)	Type of FI Indicator	Country	Households/Individuals
Thesis	Shankar-Krishnan, N. [81]	Food insecurity in Spanish adolescents: Assessment and associations with psychological wellbeing, body image and disordered eating. (2019)	9-item USDA Spanish Child Food Security Survey Module (CFSSM)	Spain	Adolescent-children
Thesis	Borowko, W [83]	Food insecurity among working-age Canadians with disabilities. (2008)	18 item USDA HFSSM	Canada	Adults
Thesis	Subnath, M [84]	Indigenous Food Insecurity in Canada: An Analysis Using the 2012 Aboriginal Peoples Survey. (2017)	6 item USDA HFSSM	Canada	Adults
Thesis	La Mantia, C [95]	Diet quality in relation to income, education and food insecurity among Canadian adults. (2020)	18 item USDA HFSSM	Canada	Adults
Thesis	Goetz, J.R [105]	Exploring food insecurity among individuals with serious mental illness: A qualitative study. (2008)	10-item USDA Adult Food Security Survey Module (AFSSM)	USA	Adults
Thesis	Stevenson, H [106]	Food security in New Zealand: The relationship between food security, ethnicity and body weight status. (2012)	8-item New Zealand Household Food Insecurity Measurement Tool	USA	Adults
Thesis	Osborne, M [117]	Gender inequality in food insecurity: An examination of single adults without children in the United States. (2012)	18 item USDA HFSSM	USA	Adults
Thesis	Ding, M [128]	Food insecurity and undiagnosed chronic conditions among adults. (2012)	18 item USDA HFSSM	USA	Adults
Thesis	Mai Dinh. A [139]	Effects of Food Insecurity on Fast Food Consumption A Cross Sectional Study. (2012)	6 item USDA HFSSM	USA	Adults
Thesis	Tayla Radean, A [150]	Investigating the link between food insecurity and disturbances in eating and mood. (2014)	6 item USDA HFSSM	USA	Adults
Thesis	Guerra S.A [161]	Does food insecurity predict depression among Brazos valley residents? (2015)	Radimer-Cornell Food Insecurity Measure	USA	Adults
Thesis	Little TV [174]	Language Proficiency, Citizenship, and Food Insecurity among Predominantly Immigrant Caribbean Latinos in Massachusetts: A Masters Thesis. (2015)	6 item USDA HFSSM	USA	Adults
Thesis	Prezzato. E.L [172]	Dietary intake and risk factors associated with food insecurity. (2015)	6 item USDA HFSSM	USA	Adults
Thesis	Rodriguez. P [173]	Development of a Food Program Design for the San Fernando Valley Rescue Mission to Improve Food Insecurity and Health Among Participants. (2016)	6 item USDA HFSSM	USA	Adults
Thesis	Mercado, V [107]	Food and housing insecurity among students at a community college district. (2017)	6-item USDA HFSSM	USA	Adults
Thesis	Kashuba, K [108]	The prevelance, correlates, and academic consequences of food insecurity among University of Oregon Students. (2017).	6 item USDA HFSSM	USA	Adults
Thesis	Eubanks, M M [109]	The Prevalence of Food Insecurity within the University of Mississippi Community and Knowledge of and Opinions About the University of Mississippi Food Bank. (2017)	6 item USDA HFSSM	USA	Adults
Thesis	Danek, A [110]	Food insecurity and related correlates among students attending Appalachian State University. (2017)	10-item USDA Adult Food Security Survey Module (AFSSM)	USA	Adults
Thesis	Harris, E. A	A socio-ecological analysis of the relationship between eating pathology and food insecurity (2018)	10 item USDA HFSSM	USA	Adults
Thesis	Hagedorn R.L [112]	WISH4Campus: Evaluating college food insecurity and promoting solutions for student wellbeing. (2019)	10 item USDA HFSSM	USA	Adults
Thesis	Sydnor, M.L [175]	Feed me, house me: Undergraduate college students’ perspectives on food and housing insecurity. (2019)	6 item USDA HFSSM	USA	Adults
Thesis	Goldson, T [114]	An examination of the impact of the supplemental nutrition assistance program on food insecurity and depression among low-income adults living in the United States (2019)	10 item USDA HFSSM	USA	Adults
Thesis	Shirley, T [115]	Food insecurity and produce behaviors of adults with children in rural, Appalachian Mississippi. (2019)	10 item USDA HFSSM	USA	Adults
Thesis	Pittman, S.E [116]	Exploring Illinois farmers’ food insecurity status. (2020)	18 item USDA HFSSM	USA	Adults
Thesis	Raja, A [119]	Food insecurity and alcohol use in people with HIV infection and substance use disorder. (2017)	HFIAS	USA	Adults
Thesis	Loopstra, R [99]	Household Food Insecurity in Canada: Towards an Understanding of Effective Interventions. (2014)	18 item USDA HFSSM	Canada	Adults and Children
Thesis	Ramsey, R [74]	Food and Brisbane households-dietary, health and social consequences of food insecurity. (2011)	18 item USDA HFSSM	Australia	Adults and Children
Thesis	Burris, M [120]	Food insecurity and age of menarche: using a biocultural approach and life history theory to assess risks of food insecurity among girls in Tampa Bay, FL. (2018)	6 item USDA HFSSM	USA	Adults and Children dyads
Thesis	Maynard, M [100]	Experiences of Food Insecurity Among Undergraduate Students at the University of Waterloo: Barriers, Coping Strategies, and Perceived Health and Academic Outcomes (2016)	18 item USDA HFSSM	Canada	College students
Thesis	Hattangadi, N [101]	Do university students who experience food insecurity report psychological distress? (2018)	6 item USDA HFSSM	Canada	College students
Thesis	Nugent, M.A [121]	Journeys to the food bank: exploring the experiences of food insecurity among postsecondary students. (2000)	18 item USDA HFSSM	USA	College students
Thesis	Maroto. M [122]	Food insecurity among community college students: prevalence and relationship to GPA, energy and concentration. (2013)	10 item USDA HFSSM	USA	College students
Thesis	Gorman, A [123]	Food insecurity prevalence among college students at Kent State University. (2014)	18 item USDA HFSSM	USA	College students
Thesis	MacDonald, A [127]	Food Insecurity and Educational Attainment at the University of Arkansas. (2016)	6-item USDA HFSSM	USA	College students
Thesis	Camelo, K [130]	Predictors of food insecurity and their relationship to academic achievement of college students. (2017).	6 item USDA HFSSM	USA	College students
Thesis	Adamovic. E [131]	Food insecurity among college students: An assessment of prevalence and solutions. (2017)	6 item USDA HFSSM	USA	College students
Thesis	Poll, K [132]	Food Insecurityand Eating Behaviors Of Collegiate Male Athletes. (2017)	18 item USDA HFSSM	USA	College students
Thesis	Kim, E [133]	The acceptability and feasibility of an on-campus food pantry to address student food insecurity. (2018)	10 item USDA HFSSM	USA	College students
Thesis	Watson, T. D [134]	Emerging topics in food insecurity: An assessment of university student food access and urban agriculture in Los Angeles. (2018)	6 item USDA HFSSM	USA	College students
Thesis	Benefield, J N [135]	Food insecurity and health of International Students. (2019)	6 item USDA HFSSM	USA	College students
Thesis	Weaver, C.G [136]	Students’ perspectives of strategies to combat food insecurity on campus. (2020)	6 item USDA HFSSM	USA	College students
Thesis	Que, S. B [137]	Food Security as a Basic Right for College Students: A Descriptive Study of the Factors Associated with Food Insecurity and Higher Education Attainment. (2020)	18 item USDA HFSSM	USA	College students
Thesis	Schichtl, R [138]	Examining the presence of empathy in college students as it relates to food insecurity. (2020)	6 item USDA HFSSM	USA	College students
Thesis	Fonseca, J. J [140]	Dietary consequences of food insecurity with Latino college students in rural America (2020)	18-item USDA HFSSM	USA	College students
Thesis	Henniger, M. M [141]	Food insecurity at an academic health campus: Estimating need, stakeholder interest, and opportunities for food assistance interventions at the university of Oklahoma health sciences center (2020)	18 item USDA HFSSM	USA	College students
Thesis	Anziano, J [142]	Food insecurity among college athletes at a public university in New England. (2020)	6 item USDA HFSSM	USA	College students
Thesis	Thirakul, N [102]	An analysis of the prevalence and predictors of food insecurity in Canadian seniors. (2019)	10 item USDA HFSSM	Canada	Elderly
Thesis	Park, J. Y [68]	Food insecurity among the elderly in developed countries: Insights from a multi-national analysis. (2019)	FIES	Developed countries	Elderly
Thesis	Carrasco, G [82]	Intervención educativa en ancianos receptores de un servicio de teleasisténcia destinado al incremento de conocimientos sobre alimentación y actividad física y a la reducción del riesgo de inseguridad alimentaria, malnutrición y sedentarismo (icaaf-rimas) (2019)	6 item USDA HFSSM	Spain	Elderly
Thesis	Robinson, H [143]	Low income older adults’ use of food pantries as a way to cope with food insecurity. (2014)	6 item USDA HFSSM	USA	Elderly
Thesis	Goodman, L. G [103]	Factors associated with food insecurity among women in a small indigenous Canadian Arctic community. (2008)	18 item USDA HFSSM	Canada	Adults-women
Thesis	Smith, J [144]	The effect of resource cycling and food insecurity on dietary intake and weight of low-income, single mothers living in rural Louisiana. (2002)	6 item USDA HFSSM	USA	Adults-women
Thesis	Yates, J. T [145]	Maternal depression and food insecurity during pregnancy among Oregon women. (2008)	2005 Oregon PRAMS single-item Food Insecurity Measure	USA	Adults-women
Thesis	Long, A [146]	The impact of food insecurity on disordered eating and impulsivity in African American women (2017)	Household Food Insecurity Access Scale (HFIAS), FAO	USA	Adults-women
Thesis	Bakar, W [72]	Measuring and exploring perspectives on food insecurity. (2010)	18 item USDA HFSSM	Australia	Households
Thesis	Kirkpatrick, S [104]	Household Food Insecurity in Canada: An Examination of Nutrition Implications and Factors Associated with Vulnerability. (2008)	18 item USDA HFSSM	Canada	Households
Thesis	McGuire, M [85]	Poverty, food insecurity and overweight/obesity in the Canadian population. (2008)	18 item USDA HFSSM	Canada	Households
Thesis	Skinner, K [86]	Prevalence and perceptions of food insecurity and coping strategies in Fort Albany First Nation, Ontario. (2013)	18 item USDA HFSSM	Canada	Households
Thesis	Calhoun, M.D [87]	Food insecurity in urban and rural settings: A mixed methods analysis of risk factors and health. (2013).	18 item USDA HFSSM	Canada	Households
Thesis	Zahariuk. S [88]	Food insecurity within the Island Lake first nation communities in Northern Manitoba, Canada. (2014)	18 item USDA HFSSM	Canada	Households
Thesis	Leroux, J [92]	Household Food Insecurity Among Older People in Canada: the Exploration of a Public Health Issue Rendered Invisible. (2018)	18 item USDA HFSSM	Canada	Households
Thesis	Mak. J [93]	Food insecurity during pregnancy in Canada. (2019)	18 item USDA HFSSM	Canada	Households
Thesis	St-Germain, A.F. [94]	Household food insecurity in Canada: Understanding the economic circumstances and policy options. (2019)	18 item USDA HFSSM	Canada	Households
Thesis	McNeill, K. I. B [75]	Talking with Their Mouths Half Full: food insecurity in the Hamilton community. (2011)	6 item USDA HFSSM	New Zealand	Households
Thesis	Fraga, M [147]	Food insecurity among first generation and non-first generation Latinx college students at CSU Stanislaus. (2002)	Modified 18-item USDA HFSSM	USA	Households
Thesis	Caesar, S.T [148]	The color of hunger: A quantitative analysis of the impact of race, class, and gender on food insecurity. (2003)	Current Population Survey Food Security Supplement (CPS FSS), USDA	USA	Households
Thesis	Cui, S [153]	Dynamics of food insecurity of families with children. (2007)	18 item USDA HFSSM	USA	Households
Thesis	Nothwehr, A [159]	Associations among food insecurity, dietary sodium and potassium intake levels, and hypertension: A cross-sectional study based on NHANES 2007–2010 data. (2014)	18 item USDA HFSSM	USA	Households
Thesis	Uber, A [160]	A Household Level Analysis Of Poverty Additionally, Food Security Characteristics During The 2007 Recession Within New York City. (2014	18 item USDA HFSSM	USA	Households
Thesis	Chappelle, N [162]	Food insecurity at Humboldt State University. (2015)	10 item USDA HFSSM	USA	Households
Thesis	Antolini, S [113]	Food insecurity and child and parent/caretaker overweight/obesity in a rural, Appalachian Mississippi Community. (2018)	10 item USDA HFSSM	USA	Households
Thesis	Shankar, K [168]	Food insecurity, race, and adolescent non-alcoholic fatty liver disease in NHANES 2001-06. (2020)	18 item USDA HFSSM	USA	Households
Thesis	Rosenberg, J [169]	Food for thought: food insecurity and academic performance. (2020)	18 item USDA HFSSM	USA	Households
Thesis	Marfo, N. Y [170]	Does Place Matter? Food Insecurity, Perceptions of Neighborhood Walkability, and Acculturation as Predictors of Treatment Outcome in an Early Childhood Obesity Prevention Study. (2019).	18 item USDA HFSSM	USA	Households (caregiver-child dyad)
Report	Innes-Hughes, C., Thrift, A., and Cosgrove, C [71]	A further analysis of the weight status and dietary characteristics of people reporting food insecurity in NSW: NSW Population Health Survey data 2007 and 2008. (2010).	A single item Food Insecurity measure	Australia	Adults
Report	El-Hajj, A., and Benhin, E (Statistics Canada) [98]	Validation of the Food Security Module in the 2018 Longitudinal and International Study of Adults. (2020)	The Longitudinal and International Study of Adults (LISA): Food Security Module	Canada	Adults
Report	Gregory, C.A., and Coleman-Jensen, A [118]	Food Insecurity, Chronic Disease, and Health Among Working-Age Adults. (2017)	10 item USDA HFSSM	USA	Adults
Report	FAO, IFAD and WFP [69]	Monitoring Food Security and Nutrition in Support of the 2030 Agenda for Sustainable Development: Taking stock and looking ahead. (2016)	FIES	Developing and Developed Countries	Adults and Children
Report	Goldrick-Rab, S., Broton, K., and Eisenberg, D [124]	Hungry to Learn: Addressing food and housing insecurity among undergraduates. Madison. (2015)	6 item USDA HFSSM	USA	College students
Report	Bedore, A [125]	Identifying food insecurity among students andconstraints for SNAP/CalFresh participation. (2016)	6 item USDA HFSSM	USA	College students
Report	Maguire, J., and O’Neill, M., and Aberson, C [126]	California State University food and housing security survey: Emerging patterns from the Humboldt State University Data. (2016).	6 item USDA HFSSM	USA	College students
Report	Goldrick-Rab S., Richardson J., and Hernandez A [176]	Hungry and Homeless in College: Results from a National Study of Basic Needs Insecurity in Higher Education. (2017)	6 item USDA HFSSM	USA	College students
Report	University of California [129]	Global Food Initiative: Food and Housing Security. (2017)	6 item USDA HFSSM	USA	College students
Report	Innes-Hughes, C., Bowers, K., King, L., Chapman, K., Eden, B [70]	Food security: The what, how, why and where to of food security in NSW. (2010)	Single-item Food Insecurity question from NSW Population Health Survey	Australia	Households
Report	Roshanafshar., S and Hawkins, E. (Statistics Canada)	Food Insecurity in Canada. Health at a Glance. (2015)	18 item USDA HFSSM	Canada	Households
Report	Li, N., Dachner, N., Tarasuk, V., Zhang, R., Kurrien, M., Harris, T., Gustin, S., and Rasali, D [90]	Priority health equity indicators for British Columbia: Household food insecurity indicator report. (2017)	18 item USDA HFSSM	Canada	Households
Report	Alberta Health Services [91]	Household food insecurity in Alberta: A backgrounder. (2017)	18 item USDA HFSSM	Canada	Households
Report	Statistics Canada [177]	Food insecurity during the COVID-19 pandemic. StatCan COVID-19: Data to Insights for a better Canada. (2020)	6 item USDA HFSSM	Canada	Households
Report	Statistics Canada [97]	Household food insecurity, 2017/2018. Health fact sheets. (2020)	18 item USDA HFSSM	Canada	Households
Report	Tarasuk, V., Mitchell A [96]	Household food insecurity in Canada, 2017-18. (2020)	18 item USDA HFSSM	Canada	Households
Report	FAO, IFAD, UNICEF, WFP and WHO [2]	The state of food security and nutrition in the world 2021	FIES	Developing and Developed Countries	Households
Report	Bates, B., Roberts, C., Lepps, H., and Porter, L [78]	The food and you survey: Wave 4. (2017)	10 item USDA HFSSM	UK	Households
Report	Loopstra. R	Vulnerability to food insecurity since the COVID-19 lockdown: Preliminary report. (2020)	18 item USDA HFSSM	UK	Households
Report	Gundersen, C., and Ribar, D. C [149]	Food Insecurity and Insufficiency at Low Levels of Food Expenditures (2005)	18 item USDA HFSSM	USA	Households
Report	Bartfeld, J., and Wang, L [151]	Local-Level Predictors of Household Food Insecurity. (2006)	6-item USDA HFSSM	USA	Households
Report	Nord, M [152]	Characteristics of low-income households with very low food security: An analysis of the USDA GPRA food security indicator. (2007)	18 item USDA HFSSM	USA	Households
Report	Nord, M., and Hopwood, A [154]	A Comparison of Household Food Security in Canada and the United States.(2008)	18 item USDA HFSSM	USA	Households
Report	Nord, M., and Golla, A.M [155]	Does SNAP Decrease Food Insecurity? Untangling the Self-Selection Effect. (2009)	modified 7-item USDA HFSSM	USA	Households
Report	Nord, M., Coleman-Jensen, A., Andrews, M., and Carlson, S [156]	Household Food Security in the United States, 2009. (2010)	18 item USDA HFSSM	USA	Households
Report	Coleman-Jensen, A., and Nord, M [157]	Food Insecurity Among Households With Working-Age Adults With Disabilities (2013)	18 item USDA HFSSM	USA	Households
Report	Nord, M., Coleman-Jensen, A., and Gregory, C [158]	Prevalence of U.S. Food Insecurity Is Related to Changes in Unemployment, Inflation, and the Price of Food. (2014)	18 item USDA HFSSM	USA	Households
Report	Gregory, C.A., Coleman-Jensen, A	Food Insecurity, Chronic Disease, and Health Among Working-Age Adults. (2017)	10 item USDA HFSSM	USA	Households
Report	Coleman-Jensen, Alisha, Matthew P. Rabbitt, Christian A. Gregory, and Anita Singh [163]	Household Food Security in the United States in 2018. (2019)	18 item USDA HFSSM	USA	Households
Report	Feeding America [165]	Map the Meal Gap 2020: A Report on County and Congressional District Food Insecurity and County Food Cost in the United States in 2018. (2020).	18-item USDA HFSSM	USA	Households
Report	Schanzenbach, D. W., and A. Pitts [166]	Food insecurity in the census household pulse survey tables. (2020)	18 item USDA HFSSM	USA	Households
Report	Coleman-Jensen, A., Rabbitt, M,P., Gregory, C.A., and Singh, A [167]	Household food security in the United States in 2019. (2020)	18 item USDA HFSSM	USA	Households
Report	Schanzenbach, D. W., and A. Pitts [178]	Estimates of food insecurity during the COVID-19 crisis: Results from the COVID impact survey, Week 1 (April 20–26, 2020). (2020)	18 item USDA HFSSM	USA	Households
Report	FAO [179]	Using the Food Insecurity Experience Scale (FIES) to monitor the impact of COVID-19. (2020)	FIES	Developing and developed countries	Households
Book chapter	Gallegos, D., Booth, S., Kleve, S., McKenchie, R., and Lindberg, R [73]	Food Insecurity in Australian Households: From Charity to Entitlement. (2017)	Single-item Food Insecurity Measure	Australia	Adolescents
Newsletter	Yau, A., Adams, J., and White, M [80]	Food insecurity in the UK—why we need a new normal. (2020)	18 item USDA HFSSM	UK	Households
Conference	Graham, P. L., Young, J., Haskell-Ramsay, C., and Fothergill, M [77]	Food insecurity and poor food habits amongst college students in the North East of England. (2020)	9-item USDA Child Food Security Survey Module CFSSM	UK	Adolescents
Conference	Furey, S., Beacom, E., McLaughlin, C., McDowell, D., and Quinn, U [79]	Comparing Food Insecurity Prevalence Using Existing Indicators. (2020)	EU Survey on Income and Living Condition, FIES (FAO) and 18-item USDA USFSSM	UK	Households
Conference	Villamor, M., Nicholson-Bell, J and Wright, L [164]	Food Insecurity as a Continuum: Investigating the Emotional Well-Being of Parents. (2020)	18 item USDA HFSSM	USA	Households

FIES: food insecurity experience scale; USDA: united states department of agriculture; LISA: the longitudinal and international study of adults.

**Table 3:** Measures of food insecurity identified in this review, and frequency of use in the different studies.

	#	%
18 item USDA HFSSM	58	38.9
6-item USDA HFSSM	34	22.8
10 item USDA HFSSM/AFSSM	15	10.1
FIES	12	8.1
HFIAS	4	2.7
Single-item Food Insecurity question from NSW Population Health Survey	3	2.0
9-item USDA Child Food Security Survey Module (CFSSM)	2	1.3
Brazilian Food Insecurity Scale	2	1.3
European Quality of Life Survey item ‘could your household afford a meal with meat, chicken or fish every second day if you wanted it?’	2	1.3
Radimer/Cornell Hunger and Food Insecurity Scale	2	1.3
2-item Food Insecurity Screening Questions (Hager et al., 2010)	2	1.3
2005 Oregon PRAMS single-item Food Insecurity Measure	1	0.7
8-item New Zealand Household Food Insecurity Measurement Tool	1	0.7
Ad hoc single item FI measure ENEP	1	0.7
Current Population Survey Food Security Supplement (CPS FSS), USDA	1	0.7
Food Insecurity Index (FII)	1	0.7
Food Security Survey Module (FSSM)	1	0.7
Healthy Diets ASAP	1	0.7
The Longitudinal and International Study of Adults (LISA): Food Security Module	1	0.7
Household Hunger Scale	1	0.7
Household Food and Nutrition Security Survey (HFNSS)	1	0.7
Girard four point tool	1	0.7
Kuyper past food insecurity	1	0.7
Townsend Food Behaviour Checklist	1	0.7

Note. #: number of times the instrument appears in the literature reviewed.

**Table 4:** Overview of measurement tools of food insecurity drawn upon in peer-reviewed articles and grey literature.

Instrument	Author/s	Year	Language	Original Purpose of Development	Unit to Collect FI Data	Time Frame of FI Assessment	Dimensions of FI	Focus on Quality/Quantity of Food	Number of Items	Response Options	Validity Support	Countries Where It Has Been Used	Respondents
HFIAS: Household Food Insecurity Access Scale (HFIAS) for Measurement of Food Access	FAO	2007	EN,ES,FR	The Household Food Insecurity Access Scale (HFIAS) provides a simple and user-friendly approach for measuring the impacts of development food aid programs on the access component of household food insecurity across different cultural contexts. It is to be used especially in resource-poor areas. The information generated by the HFIAS can be used to assess the prevalence of household food insecurity (access component) and to detect changes in the food insecurity situation of a population over time.	Household	4 weeks	Access	Quantity	9 occurrence + 9 frequency questions	Dichotomous (occurrence questions) Three-option ordinal (frequency questions), which are translated into four-option responses for each of the nine questions	YesYes in developing countries—although used universally	Mostly low-income (but selected references are from US)	All included (household approach)
FIES: Food Insecurity Experience Scale	FAO	2013	EN, ES, FR and Arabic Translations provided for more than 170 languages and dialects	The FIES can be used to measure food security across sociocultural contexts for the following purposes: To assess the population prevalence of food insecurity (for both SDG monitoring and national use); To identify vulnerable populations; To guide and monitor the effects of food security policies and programs; To identify risk factors and consequences of food insecurity	Individual (but applicable to household if the HH version is selected)	30 days or 12 months	Access	Quantity	8	Dichotomous	Yes	Globally (all-income countries)	Adults, elderly, households (includes children)
18-item USDA HFSSM	USDA	first implemented in 1995; last revised in 2012	EN, ES, ZH, FA	Initially, the tool was intended to measure both household food insecurity and hunger. The USDA made few changes. They decided to omit hunger as it is a physiological concept that cannot be measured. Further, changes were made in the food insecurity labels (food secure, low food security and very low food security).	Household	30 days or 12 months	Access	Mostly quantity—Only two questions focus on balanced meal and low-cost food	18	Multiple choice of three stages with screeners and statements and dichotomous variables.	Yes	USA, Canada, UK, Australia	Children, adolescents, adults including elderly
10-item USDA HFSSM or AFSSM (Adult Food Security Survey Module)	USDA	The AFSSM is based on the 18-item US HFSSM implemented in 1995 and revised in 2012.	EN, ES	Same as the other HFSSM	Adults	30 days or 12 months	Access	Mostly quantity, with some questions about quality	10	Multiple choice with 3 stages and screeners and dichotomous variables	Yes	USA, Canada, UK	Adults
9-item USDA Child Food Security Survey Module (CFSSM)	USDA	The CFSSM was adapted from the 18-item HFSSM and developed by researchers Connell et al., 2004.	EN, ES	Same as the other HFSSM	Children (adolescents) aged 12 years and older	30 days or 12 months	Access	Mostly quantity, with some questions about quality	9	Multiple choice with statements.	Yes	USA, UK, Spain	Adolescents
6-item USDA HFSSM	USDA	The questions are unchanged from the survey implemented in 1995 and developed by Blumberg et al., 1999.	EN	Same as the other HFSSM	Household and individual	30 days or 12 months	Access	Mostly quantity, with some questions about quality	6	Multiple choice with statements.	Yes	USA, Canada, UK, New Zealand, Spain	Adults (individual or household)
Household Hunger Scale (HHS)	FAO/FANTA	2011	EN, ES, FR	This questionnaire departs from HFIAS in order to develop a tool that is applicable to identify food insecure households cross-cultural settings in developing countries.	Household	4 weeks	Access	Quantity	3 occurrence + 3 frequency questions	Dichotomous (occurrence questions) Three-option ordinal (frequency questions), which are translated into four-option responses for each of the three questions	Yes for seven diverse contexts in developing countries, but used globally	Europe	Household
Single item Australia	NSW Population Health Survey	2007	EN	This single item of FI was included in the NSW Population Health Survey along with a series of other health related questions	Household of adults aged 16 years and older	12 months	Access	Quantity	1	Dichotomous. Food insecure or Food secure	YesNoNo	Australia	Adults (16 years and older)
LISA Food Security Module	Statistics Canada	2020	EN	To identify the prevalence and correlates of food insecurity across population groups	Household, individuals (adults and children)	12 months	Access	Mostly quantity, with some questions about quality	18	Response options are same as HFSSM. The modified household scale has an additional FI status, i.e., food insecure marginal	Yes	Canada	Adults and children
8-item New Zealand Household Food Insecurity measurement tool	Parnell and Gray, 2014	Initial work in 1997 by Reid but final version developed by Parnell and Gray in 2014	EN	To identify the prevalence and correlates of food insecurity across population groups	Household	12 months	Access	Mostly quantity, with some questions about quality	8	Polychotomous.	Yes	New Zealand	Households
Radimer-Cornell Food Insecurity Measure	Radimer et al.	1990	EN, Malay, Farsi, French	The first pathway dealt with insufficient intake of food and food restriction, along with the physical feelings associated with being hungry. The second pathway included household problems with food supply, diet quality, feelings regarding the household food situation, and what the household did to alleviate issues with lack of food	Household	The instrument does not use a specific reference period	Access	Mostly quantity, with some questions about quality	There are several versions of this instrument which have been shortened including a 2-item version and a single-item version.	Dichotomous	Yes	USA	Households
2-item Food Insecurity Screening Questions (Hager et al., 2010)	Hager et al., 2010	2010	EN, ES	This instrument is the validated and modified version of the USDA’s 18-item HFSSM. Considering that the 18-item HFSSM is long and time consuming, the authors decided on modifying the original instrument to include 2 items.	Household	12 months	Access	Quantity	2	Dichotomous	Yes	USA	Households
2005 Oregon PRAMS single-item Food Insecurity Measure	Oregon Prams Survey	2005	EN	The PRAMS survey was developed for households with mothers and children. It was a survey used to capture maternal depression and household FI.	Household (mothers and babies)	12 months	Access	Quantity	1	Dichotomous	N/A	USA	Households (mothers and babies)
Brazilian Food Insecurity Scale (EBIA)	IBGE	2004 (validation)	Portuguese, English, Guarani	To identify the prevalence and severity of food insecurity	Household	Different reference periods including 1 month, 3 months, etc.	Access	Mostly quantity, with some questions about quality	14	Dichotomous	Yes	Portugal, Brazil	Households
Community Childhood Hunger Identification Project	Wehler et al.	2004	English	Part of a survey instrument to examine the prevalence of hunger among low-income families.	Household	12 months	Access	Quantity	13	Dichotomous	Yes	USA, Canada, New Zealand and England	Usually households though it has also been used to measure FI in individuals (women)
Household Food and Nutrition Security Survey (HFNSS)	Archer et al. (development of survey)	2014	English	The HFNSS was developed using a series of focus groups and a three-stage Delphi survey.	Household	12 months	Access	Quantity and quality	26	Dichotomous	Yes	Australia	Households
Girard four point tool	Girard and Sercia	2013	English	It was developed by Girard and Sercia to measure changes in food habits of first-generation immigrants in Montreal.	Household and individual	Last few weeks	Access	Quantity and financial constraints	4	Dichotomous	Yes	Canada	Households
Kuyper past food insecurity	Kuyper et al.	2006	English	The purpose of this survey was to examine whether past and current food insecurity influence child feeding practices, especially those less restrictive or more indulgent practices	Household	It asks questions about FI since childhood	Access and stability	Neutral	7	Dichotomous	Yes	USA	Households
Townsend Food Behaviour Checklist	Townsend et al.	2003	English	The purpose of this survey was to assess whether the respondent was consuming enough food based on a checklist of healthy foods. It included two FI questions which asks the respondent whether they have enough to eat	Individual	Before the end of the month	Access	Neutral	2	Ordinal: Four-point categorical scale	Yes	USA	Individuals
Pre-program questionnaire single item FI measure ENEP	ENEP	N/A	English	It employs a single item FI measure which is a part of a food literacy behaviour checklist	Individual	Past month	Access	Quantity	1	N/A	N/A	USA	Individuals
European Quality of Life Survey (EQLS)	European Foundation for the Improvement of Living and Working Conditions (Eurofoun)	2003, 2007	English	It focuses on an aspect of food consumption (meat/chicken/fish) that families economise on during periods of financial strain, and is less subjective than other measures	Household	12 months	Access	Quantity	1	Dichotomous.	N/A	Europe	Individuals
Food Insecurity Index	Bacon and Baker	2017	English	To identify the percentage of individuals within a census tract who are food insecure and its correlates	Household	N/A	Access	N/A	N/A	N/A	N/A	USA	Household
Healthy Diets ASAP	Lee et al., 2018	2018	English	The Healthy Diets ASAP methods protocol was developed to assess, compare and monitor the price and affordability of healthy and current diets among the general population in Australia.	Household	Fortnightly recommendation of healthy diet	Availability and acess	Quantity	N/A	N/A	Yes	Australia	Household

## Data Availability

The data presented in this study are available on request from the corresponding author.

## Supplementary Materials

The following are available online at https://www.mdpi.com/article/10.3390/ijerph18189829/s1, Supplementary file S1: search strategies.
