# Supplementary material for: The Measurement of Food Insecurity in High-Income Countries: A Scoping Review
Source: Int J Environ Res Public Health. 2021 Sep 17;18(18):9829. doi: 10.3390/ijerph18189829 (PMC8468034; doi:10.3390/ijerph18189829)
Supplement: Supplementary file 1 [file ijerph-18-09829-s001.zip › ijerph-1340765-supplementary.pdf]

CINAHL. (10 results)
("food insecurity" OR "food security" OR "dietary diversity") AND ("measure*" OR "scale" OR "questionnaire" OR "assessment") AND ("high income countr*" OR "developed countr*) AND ("individual*" OR "household*")
From *2000/1/1* to *2020/12/31*
Age groups: *adolescent: 13-18 years* + *all adult* + *adult: 19-44 years* + middle aged: 45-64 years + aged: 65+ years
Apply related words + apply equivalent topics

COCHRANE TRIALS. (7 results)
("food insecurity" OR "food security" OR "dietary diversity") AND (measure* OR scale OR questionnaire OR assessment) AND ("high income countr*" OR "developed countr*) AND (individual OR household*)
Publication Year from 2000 to 2020, in Trials
(Word variations have been searched)

FSTA (Food Science). (34 results)
(food insecurity.mp. OR food security.mp. OR dietary diversity.mp.) AND (measure*.mp. OR scale$.mp. OR questionnaire$.mp. OR assessment$.mp.) AND (developed countr*.mp. OR high income country.mp. OR high income countries.mp.) AND (individual$.mp. OR household$.mp.)
Publication date: 2000-2020.

MEDLINE (PubMed). (17 results)
((("food insecurity" OR "food security" OR "dietary diversity") AND ("measure*" OR "scale*" OR "questionnaire*" OR "assessment*")) AND ("high income countr*" OR "developed countr*")) AND ("individual*" OR "household*")
From *2000/1/1* to *2020/12/31*
Age: From *Adolescent* to *80 and over*

PSYCINFO. (6 results)
("food insecurity" OR "food security" OR "dietary diversity") AND ("measure*" OR "scale" OR "questionnaire" OR "assessment") AND ("high income countr*" OR "developed countr*) AND ("individual*" OR "household*")
Publication date: 20000101-20201231
Age: From Adolescents to Very Old
Apply related words + apply equivalent topics

SCOPUS. (66 results)
(TITLE-ABS-KEY (("food insecurity" OR "food security" OR "dietary diversity")) AND TITLE-ABS-KEY ("measure" OR "measurement" OR "scale" OR "questionnaire" OR "assessment") AND TITLE-ABS-KEY ("high income country" OR "developed country) AND TITLE-ABS-KEY ("individual" OR "household"))
From *2000/1/1* to *2020/12/31*

SOCIOLOGICAL ABSTRACTS. (3 results)
noft("food insecurity" OR "food security" OR "dietary diversity") AND noft(measure OR scale OR questionnaire OR assessment) AND noft("high income country" OR "developed country") AND noft(individual OR household)
Include: Spelling variants of search terms + Form variants of your search terms
noft=*anywhere except full text*
Date: From 01 January 2000 to 31 December 2020

WOS (Web Of Science Core Collection). (Results: 59)
TOPIC: ("food insecurity" OR "food security" OR "dietary diversity") *AND* TOPIC: (measure* OR scale* OR questionnaire* OR assessment*) *AND* TOPIC: ("high income countr*" OR "developed countr*) *AND* TOPIC: (individual* OR household*)

Timespan: 2000-2020. Search language=Auto
